# Recognition of DNA Methylation Molecular Features for Diagnosis and Prognosis in Gastric Cancer

**DOI:** 10.3389/fgene.2021.758926

**Published:** 2021-10-21

**Authors:** Donghui Liu, Long Li, Liru Wang, Chao Wang, Xiaowei Hu, Qingxin Jiang, Xuyao Wang, Guiqin Xue, Yu Liu, Dongbo Xue

**Affiliations:** ^1^ Department of Oncology, Heilongjiang Provincial Hospital, Harbin, China; ^2^ Harbin Institute of Technology, School of Life Science and Technology, Harbin, China; ^3^ Department of General Surgery, First Affiliated Hospital of Harbin Medical University, Harbin, China; ^4^ Key Laboratory of Hepatosplenic Surgery, Ministry of Education, The First Affiliated Hospital of Harbin Medical University, Harbin, China; ^5^ Department of Cardiology, Second Affiliated Hospital of Harbin Medical University, Harbin, China; ^6^ Department of Head and Neck and Genito‐Urinary Oncology, Harbin Medical University Cancer Hospital, Harbin, China; ^7^ Department of General Surgery, Harbin 242 Hospital of Genertec Medical, Harbin, China; ^8^ Department of Pharmacy, Harbin Second Hospital, Harbin, China; ^9^ Department of General Surgery, Daqing Fifth Hospital, Daqing, China; ^10^ Department of Endocrine, Heilongjiang Provincial Hospital, Harbin, China

**Keywords:** gastric cancer, tumor marker, diagnosis, prognosis, DNA methylation, mutation

## Abstract

**Background:** The management of gastric cancer (GC) still lacks tumor markers with high specificity and sensitivity. The goal of current research is to find effective diagnostic and prognostic markers and to clarify their related mechanisms.

**Methods:** In this study, we integrated GC DNA methylation data from publicly available datasets obtained from TCGA and GEO databases, and applied random forest and LASSO analysis methods to screen reliable differential methylation sites (DMSs) for GC diagnosis. We constructed a diagnostic model of GC by logistic analysis and conducted verification and clinical correlation analysis. We screened credible prognostic DMSs through univariate Cox and LASSO analyses and verified a prognostic model of GC by multivariate Cox analysis. Independent prognostic and biological function analyses were performed for the prognostic risk score. We performed TP53 correlation analysis, mutation and prognosis analysis on eleven-DNA methylation driver gene (DMG), and constructed a multifactor regulatory network of key genes.

**Results:** The five-DMS diagnostic model distinguished GC from normal samples, and diagnostic risk value was significantly correlated with grade and tumor location. The prediction accuracy of the eleven-DMS prognostic model was verified in both the training and validation datasets, indicating its certain potential for GC survival prediction. The survival rate of the high-risk group was significantly lower than that of the low-risk group. The prognostic risk score was an independent risk factor for the prognosis of GC, which was significantly correlated with N stage and tumor location, positively correlated with the VIM gene, and negatively correlated with the CDH1 gene. The expression of CHRNB2 decreased significantly in the TP53 mutation group of gastric cancer patients, and there were significant differences in CCDC69, RASSF2, CHRNB2, ARMC9, and RPN1 between the TP53 mutation group and the TP53 non-mutation group of gastric cancer patients. In addition, CEP290, UBXN8, KDM4A, RPN1 had high frequency mutations and the function of eleven-DMG mutation related genes in GC patients is widely enriched in multiple pathways.

**Conclusion:** Combined, the five-DMS diagnostic and eleven-DMS prognostic GC models are important tools for accurate and individualized treatment. The study provides direction for exploring potential markers of GC.

## Introduction

According to the statistics released by the World Health Organization in 2018, the incidence and mortality rate of gastric cancer (GC) ranked fifth and third, respectively, among cancers worldwide. GC is a characteristic cancer in East Asia with an incidence rate of 32.1/100,000 and a mortality rate of 13.2/100,000 (1). Among Eastern Asian countries, Japan, South Korea, and China have the highest GC morbidity and mortality rates in the world (Bray et al., 2018). Therefore, the prevention and treatment of GC are essential for improving patient outcomes. Although advances in surgery, radiotherapy, chemotherapy, molecular targeting, and immunotherapy have improved overall prognosis, diagnosis of GC is often delayed, resulting in unsatisfactory outcomes (Bang et al., 2017; Cats et al., 2018; Sundar et al., 2019). It is, thus, urgent to explore effective biomarkers for early diagnosis and prognosis prediction of GC.

Epigenetic markers have been widely recognized in recent years, particularly promoter hypermethylation. Compared with a wide range of mutational variations in a specific gene, promoter hypermethylation occurs in the same defined region of a gene in all forms of cancer (Fu, 2015). Therefore, diagnosis and prognosis prediction of patients with GC can be reliably obtained at the epigenetic level *via* differential expression of common DNA methylation (DNAm). DNAm is a major epigenetic modification that participates in many important life activities, such as cell proliferation, differentiation, development, apoptosis, tumor development, and occurrence of other diseases, and it is also one of the earliest discovered DNA modifications. DNAm can cause changes in chromatin structure and DNA stability, thereby regulating gene expression (Neri et al., 2017). Abnormal DNAm located in the promoter region usually leads to silencing of tumor suppressor genes or high expression of proto-oncogenes, thereby promoting tumor progression (Das and Singal, 2004). Among them, hypermethylation of tumor suppressor genes is the most common and can be used as an early tumor marker. Some specific DNAm sites are closely related to GC, such as cell cycle-related genes P16 and MDGA2 (Hibi et al., 2003; Wang et al., 2016), tumor suppressor genes, apoptosis-related genes PCDH10 and BCL6B (Yu et al., 2009; Xu et al., 2012), signal transduction-related genes FOXF2 and RUNX3 (Sakakura et al., 2005; Higashimori et al., 2018), and proto-oncogenes RAS and c-myc (Nishigaki et al., 2005; Licchesi et al., 2010). The discovery of these DNAm sites has broad application value in the early diagnosis, prognosis, and even treatment of GC. However, only a small number of DNAm sites have been approved for use as basic tumor markers (NDRG4, BMP3, and SEPTIN9) (Imperiale et al., 2014; FDA). There are many reasons for this, such as small numbers of test samples, patient selection bias, lagging research design and data analysis methods, lack of substantial clinical value, and other factors have prevented thorough evaluation of the clinical value of GC biomarkers. With the development of bioinformatics, enabling the establishment of GC diagnostic and prognostic models based on big data, the above problems can be resolved.

Few studies have described the application of a differential methylation site (DMS) scoring system to construct individualized GC diagnostic and prognostic models. In this study, we integrated publicly available GC DNAm datasets obtained from The Cancer Genome Atlas (TCGA) and Gene Expression Omnibus (GEO) databases to construct a diagnostic model and verify its ability to distinguish GC from normal tissues. The DMSs were then matched with overall survival (OS) data and a prognostic model was constructed. Finally, the prognostic model was analyzed to explore its clinical application and potential molecular mechanisms in patients with GC. The correlations between clinical correlation analysis of the diagnostic and analysis of independent prognostic factors will help achieve accurate and individualized treatment in a clinical setting.

## Materials and Methods

### Obtaining DNAm Data of Gastric Cancer

We downloaded TCGA GC DNAm profiles (Illumina Human Methylation 450 BeadChip, Illumina Human Methylation 27 BeadChip), expression profiles, and corresponding clinical data through the UCSC Xena database (https://xena.ucsc.edu/) (Wang et al., 2019). The Illumina Human Methylation 450 BeadChip DNAm dataset contained two normal samples and 395 GC samples, while the Illumina Human Methylation 27 BeadChip DNAm dataset contained 25 normal samples and 48 GC samples. The expression profile dataset contained 32 normal samples and 372 GC samples. Table 1 lists the clinicopathological characteristics of the patients with GC. We downloaded the GC DNAm profile dataset GSE30601 from the GEO database (https://www.ncbi.nlm.nih.gov/geo/) (Kurashige et al., 2016). The GSE30601 dataset was based on the GPL8490 platform (Illumina Human Methylation 27 BeadChip), containing 94 normal samples and 203 GC samples. The data from TCGA GC DNAm profiles were sorted and merged as the training dataset; the GEO GC DNAm profile dataset was used as the validation dataset. Because of the availability of public data in TCGA and GEO databases, this study did not require ethical approval or informed consent.

**TABLE 1:** The clinicopathological characteristics of GS patients.

	Alive (*n* = 216)	Dead (*n* = 107)	Total (*n* = 323)
Gender			
FEMALE	89 (41.2%)	34 (31.8%)	123 (38.1%)
MALE	127 (58.8%)	73 (68.2%)	200 (61.9%)
Age			
Mean (SD)	63.9 (10.7)	65.8 (10.3)	64.5 (10.6)
Median [MIN, MAX]	65 [30,90]	67 [41,90]	66 [30,90]
Grade			
G1	5 (2.3%)	2 (1.9%)	7 (2.2%)
G2	74 (34.3%)	34 (31.8%)	108 (33.4%)
G3	137 (63.4%)	71 (66.3%)	208 (64.4%)
Stage			
Stage I	30 (13.9%)	8 (7.5%)	38 (11.8%)
Stage II	84 (38.9%)	26 (24.3%)	110 (34.0%)
Stage III	94 (43.5%)	62 (57.9%)	156 (48.3%)
Stage IV	8 (3.7%)	11 (10.3%)	19 (5.9%)
T			
T1	13 (6.0%)	1 (0.9%)	14 (4.3%)
T2	41 (19.0%)	16 (15.0%)	57 (17.6%)
T3	106 (49.1%)	56 (52.3%)	162 (50.2%)
T4	56 (25.9%)	34 (31.8%)	90 (27.9%)
M			
M1	209 (96.8%)	99 (92.5%)	308 (95.4%)
M2	7 (3.2%)	8 (7.5%)	15 (4.6%)
N			
N0	84 (38.9%)	24 (22.4%)	108 (33.5%)
N1	52 (24.1%)	30 (28.0%)	82 (25.4%)
N2	42 (19.4%)	25 (23.4%)	67 (20.7%)
N3	38 (17.6%)	28 (26.2%)	66 (20.4%)
Race			
ASIAN	63 (29.2%)	21 (19.6%)	84 (26%)
BLACK	3 (1.4%)	6 (5.6%)	9 (2.8%)
WHITE	150 (69.4%)	80 (74.8%)	230 (71.2%)
Position			
Body of stomach	54 (25%)	18 (16.8%)	72 (22.3%)
Cardia, NOS	49 (22.7%)	29 (27.1%)	78 (24.1%)
Fundus of stomach	33 (15.3%)	14 (13.1%)	47 (14.6%)
Gastric antrum	77 (35.6%)	40 (37.4%)	117 (36.2%)
Stomach, NOS	3 (1.4%)	6 (5.6%)	9 (2.8%)

### Identification of Differential Methylated Sites

We performed background correction and normalization on the DNAm data in the training set (Zhang et al., 2019). Using normal samples as controls, we screened the DMSs in GC samples using the Wilcoxon test (Xu et al., 2017), with |log2 fold change (FC)| > 1 and false discovery rate (FDR) < 0.01 set as the threshold considered to have biological significance. The “pheatmap” package in R software was used to draw a DNAm heatmap of DMSs in GC.

### Screening of Diagnostic DNAm Markers

We used the random forest method in R software to predict key DNAm sites in GC. The DNAm sites were sorted from high to low according to their calculated “Mean Decrease Accuracy” value, and 10-fold cross validation was performed five times to screen representative DNAm markers in GC. We also used the “glmnet” package in R software to predict key DNAm sites in GC through LASSO regression analysis. DMSs that could distinguish tumors from normal samples were defined as representative DNAm markers in GC. Finally, shared DNAm markers predicted by both methods were selected as reliable DNAm markers for GC diagnosis (Zhou et al., 2019a).

### Construction of DNAm Diagnostic Model

The “glm” package in R software was used to construct a diagnosis prediction model with five reliable DNAm markers through multivariate logistic regression analysis. The constructed GC diagnosis prediction model was applied to distinguish GC from normal samples in the training and validation datasets, and the model’s accuracy was evaluated. Unsupervised hierarchical clustering was used to show the DNAm status of five credible diagnostic DNAm markers in the training set and validation set.

### Correlation Analysis of DNAm Diagnostic Model With Clinical Indicators

To evaluate the clinical application of the DNAm diagnostic model in GC, we calculated the scores of patients with GC in TCGA dataset using the constructed DNAm diagnostic model. Samples with missing clinical characteristics were removed, and correlations between diagnostic score and clinical characteristics of patients were analyzed. The t-test was used for comparisons between two groups, and the Kruskal–Wallis test was used for comparisons between two or more groups. *p* < 0.05 was considered statistically significant.

### Construction of Prognostic Model Based on Differential Methylated Sites

The “survival” package in R software was used to determine DNAm sites of differential methylation associated with survival of patients with GC through univariate Cox regression analysis, and the random forest map was plotted for the top 20 DNAm sites with the most significant differences (*p* < 0.01). Based on the selected prognosis-related DNAm sites, the “glmnet” package in R software was used to perform 10,000 simulations through LASSO regression analysis, and key DNAm sites were obtained after removing overlap through cross validation.

We used multivariate Cox regression analysis to construct the following risk score formula for each patient (cg07990939 Methylation levels*(−8.908))+(cg08317263 Methylation levels*(−1.739))+(cg10301990 Methylation levels *(−4.088))+(cg10968649 Methylation levels *(−20.267))+(cg13801416 Methylation levels *(−1.009))+(cg19614321 Methylation levels*(−1.779))+(cg20074795 Methylation levels *(12.778))+(cg21052164 Methylation levels *(−0.941))+(cg26069252 Methylation levels *(7.734))+(cg26089280 Methylation levels *(−8.569))+(cg27662379 Methylation levels *(−7.672)). Patients were divided into low-risk and high-risk groups according to the risk score formula using the median risk as the cut-off point. We assessed survival differences between the two groups using the Kaplan–Meier method, and compared these survival differences using log-rank statistics. Receiver operating characteristic (ROC) curve analysis was used to determine the accuracy of model predictions (Xu et al., 2017).

### Analysis of Independent Prognostic Factors and Prognostic Risk Model

To evaluate the prognostic model and the effect of different clinical characteristics of patients with GC on prognosis and survival, we obtained phenotypic information of all samples from the clinical data in TCGA dataset and extracted the risk model samples separately, as well as the corresponding age, gender, and other phenotypic and clinical information. We combined the information in the risk model with the survival status of patients, then used the “survival” package in R software to perform univariate and multivariate independent prognostic analyses to test the ability of the prognostic risk model and the clinical characteristics of patients with GC to predict the prognosis (Vasiljević et al., 2014).

### Functional Analysis of Prognostic Risk Score

To evaluate the clinical application and important functions of the DNAm prognostic model in GC, we first calculated the risk scores of patients with GC in TCGA dataset using the constructed DNAm prognostic model and combined the risk scores with their clinical data. Samples with missing clinical traits were removed, and the correlation between risk scores and clinical characteristics was analyzed. We used the t-test to compare two groups and the Kruskal–Wallis test to compare two or more groups. *p* < 0.05 was considered statistically significant. We then extracted the expression levels of regulatory, cytotoxic, and epithelial–mesenchymal transition (EMT) factors of known immune checkpoint sites from the GC samples in TCGA dataset and correlated these levels with the risk scores of these samples to investigate whether the risk scores played an important regulatory role in GC by influencing the above factors. Finally, patients were divided into low-risk and high-risk groups according to the prognostic risk model using the median risk as the cut-off point. The low-risk group was used as the control. We used the Wilcoxon test to screen significant differentially expressed genes in the high-risk group, using the standard threshold |log2FC| > 0 and FDR <0.05. The “clusterProfiler” package in R language was used to perform gene set enrichment analysis (GSEA) for the potential mechanism of c2 (c2.cp.kegg.v7.1.entrez.gmt, c2.cp.biocarta.v7.1.entrez.gmt) and c5 (c5.bp.v7.1.entrez.gmt) in the molecular signature database (MSigDB). The number of random sample arrangements was set to 1,000, and the significance threshold was set to *p* < 0.05 (Zhou et al., 2019a).

### Analysis of the Correlation Between Eleven Prognostic-Related DMG and TP53 Mutations

UALCAN (http://ualcan.path.uab.edu/analysis.html) is a comprehensive, user-friendly and interactive online data analysis website based on relevant cancer data found in TCGA database. We used the UALCAN database to evaluate the expression levels of eleven prognostic-related DMG in gastric cancer and normal gastric tissues (Chandrashekar et al., 2017). Considering the unequal variances, the significance of differences in the transcriptional levels was evaluated using the Student’s t-test, and a *p* value of <0.05 was considered statistically significant.

### Mutation and Prognostic Analysis of Eleven Prognostic-Related DMG

The cBioPortal (http://www.cbioportal.org) integrates data from large-scale cancer research projects, such as TCGA and the International Cancer Genome Consortium (ICGC), whose gene data types cover somatic mutations, DNA copy number changes, mRNA and microRNA expression, DNA methylation, protein and phosphorus protein abundance, and provides visual and multidimensional cancer genomic data (Cerami et al., 2012; Gao et al., 2013). This study based on TCGA database, gene expression data of 412 GC patients were included. We obtained the relevant module information about 11-DMG mutation from the cBioPortal. Set the parameters: “Enter a z-score threshold±1.8”, then enter DMG to generate a mutation frequency visualization chart, and then select the top 10 genes significantly related to each gene mutations in “Co-expression” module, delete duplicates and import them into Metascape. Metascape (https://metascape.org/gp/index.html#/main/step1) is a gene list analysis tool. It integrates data from over 40 types of biological information databases for gene annotation and analysis, and provides a unique protein–protein interaction (PPI) network analysis function. We used the “Multiple Gene list” module of the Metascape tool to perform gene annotation and enrichment analyses on the genes obtained from the cBioPortal that were highly related to DMG mutations(27), and set the parameters: “enrichment factor Min overlap = 3,” “*p*-value cut-off value <0.01,” “Min enrichment >1.5” is considered statistically significant, then select Gene Ontology (GO) enriching “Biological Processes,” “Cellular Components” and “Molecular Functions” and “KEGG pathways” classification. To further capture the relationships between the terms, a subset of enriched terms was selected and rendered as a network plot, where terms with a similarity >0.3 were connected by edges. We selected the terms with the best *p*-values from each of the 20 clusters, with the constraint that there were no more than 15 terms per cluster and no more than 250 terms in total. The network was visualized using Cytoscape (Shannon et al., 2003), where each node represented an enriched term and was colored first by its cluster ID, and then by its *p*-value. For each given gene list, PPI enrichment analysis was carried out using the following databases: STRING (Szklarczyk et al., 2019), BioGrid (Oughtred et al., 2019), OmniPath (Li et al., 2017), and InWeb_IM (Li et al., 2017). Only physical interactions in STRING (physical score >0.132) and BioGrid were used (details). The molecular complex detection (MCODE) algorithm (Bader and Hogue, 2003) was applied to identify densely connected network components.

### Construction of Multi-Factor Regulatory Network of Key Genes

In order to predict the regulatory factors of key genes related to the prognostic model constructed in gastric cancer, we predicted the upstream regulated miRNAs of key genes through Starbase (http://starbase.sysu.edu.cn/index.php) and TargetScan (http://www.targetscan.org/vert_71/), and intersected the prediction results to obtain reliable miRNAs. After that, we further predicted the lncRNA upstream regulated by the trusted miRNA through the Starbase database, and predicted the transcription factors (TF) that can regulate key genes through the TRRUST (https://www.grnpedia.org/trrust) database. Finally, the regulatory network among mRNA, miRNA, lncRNA and TF was constructed by Cytoscape v3.6.1 software.

## Results

### Identification of Differential Methylated Sites in Gastric Cancer

**FIGURE 1:**
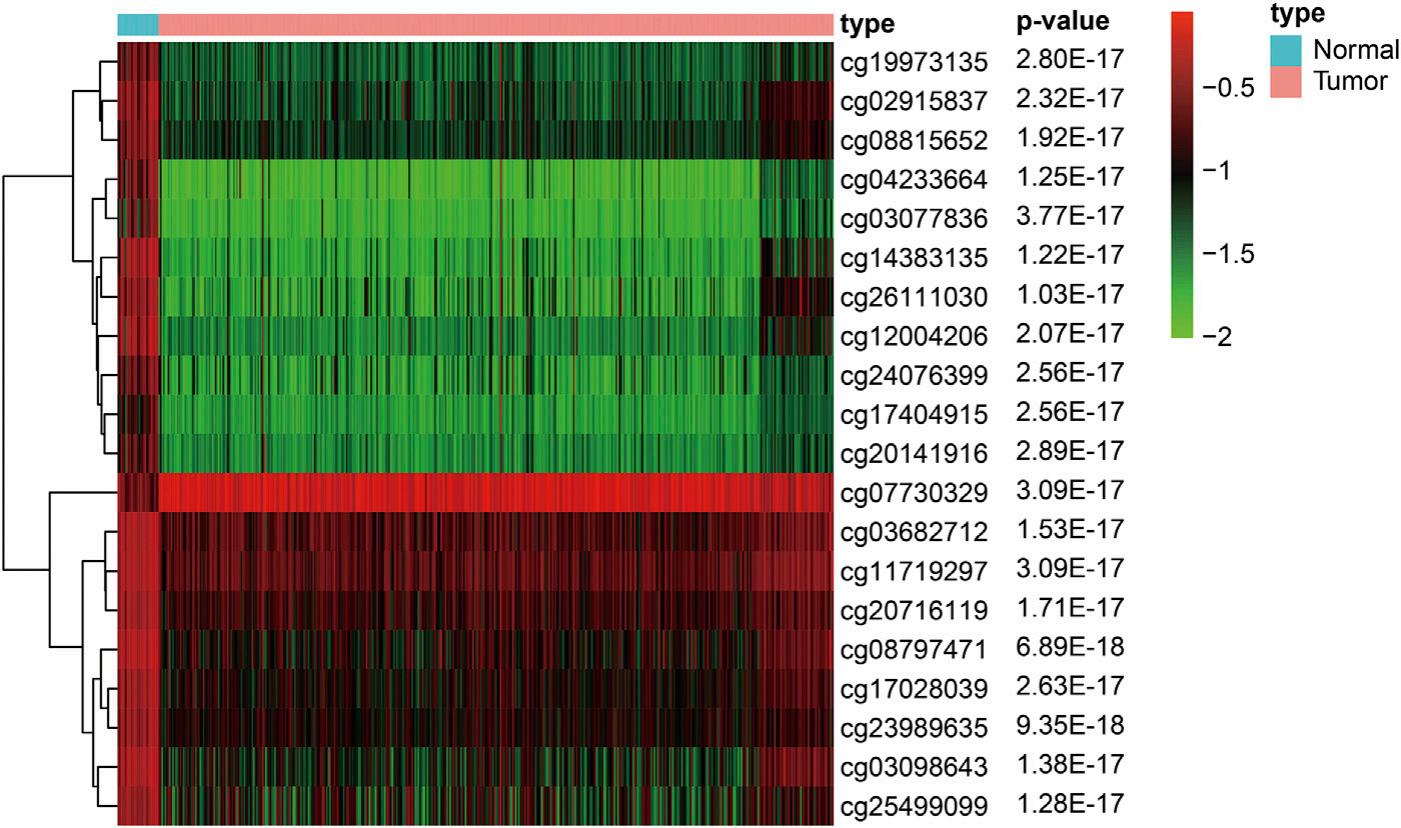
Heat map of the top 20 significanly different methylation sites in gastric cancer (Arranged in *p*-value order).

To construct the diagnostic and prognostic GC models, we performed background correction and normalization on the DNAm data from 27 normal samples and 443 GC samples in the training dataset. Among them, 1842 hypermethylated and 899 hypomethylated sites were screened out in the GC samples. We used R software package pheatmap to draw the methylation heat map of the top 20 significanly different methylation sites in gastric cancer, arranged in *p*-value order (Figure 1) (Supplementary Table S1).

### Screening of Diagnostic DNAm Markers

**FIGURE 2:**
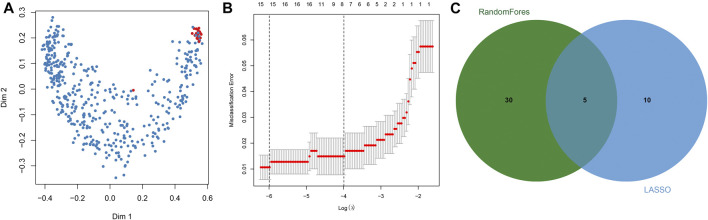
Screening of diagnostic DNA methylation (DNAm) markers in gastric cancer. **(A)** Multi-dimensional scaling plot of the proximity matrix generated from random forest analysis in the training dataset. Red dots represent normal samples and blue dots indicate tumor samples. **(B)** Misclassification error for different numbers of variables revealed by the LASSO regression model. Red dots represent the value of misclassification error, grey lines represent the standard error (SE), the two vertical dotted lines on the left and right represent optimal values by the minimum and 1-SE criteria, respectively. “Lambda” is the tuning parameter. **(C)** Screening of DNAm markers for reliable diagnosis. The green circle represents representative DNAm markers selected by random forest analysis, and the blue circle indicates representative DNAm markers screened by LASSO regression analysis.

Key DNAm sites in GC were predicted through random forest analysis, combined with five repeated ten-fold cross validations, resulting in 35 representative DNAm markers (Figure 2A). At the same time, we also predicted 15 key DNAm sites in GC by LASSO regression analysis (Figure 2B). The intersection of the representative DNAm markers predicted by both methods yielded five reliable diagnostic DNAm markers in GC (Figure 2C).

### Construction of a DNAm Diagnostic Model

**FIGURE 3:**
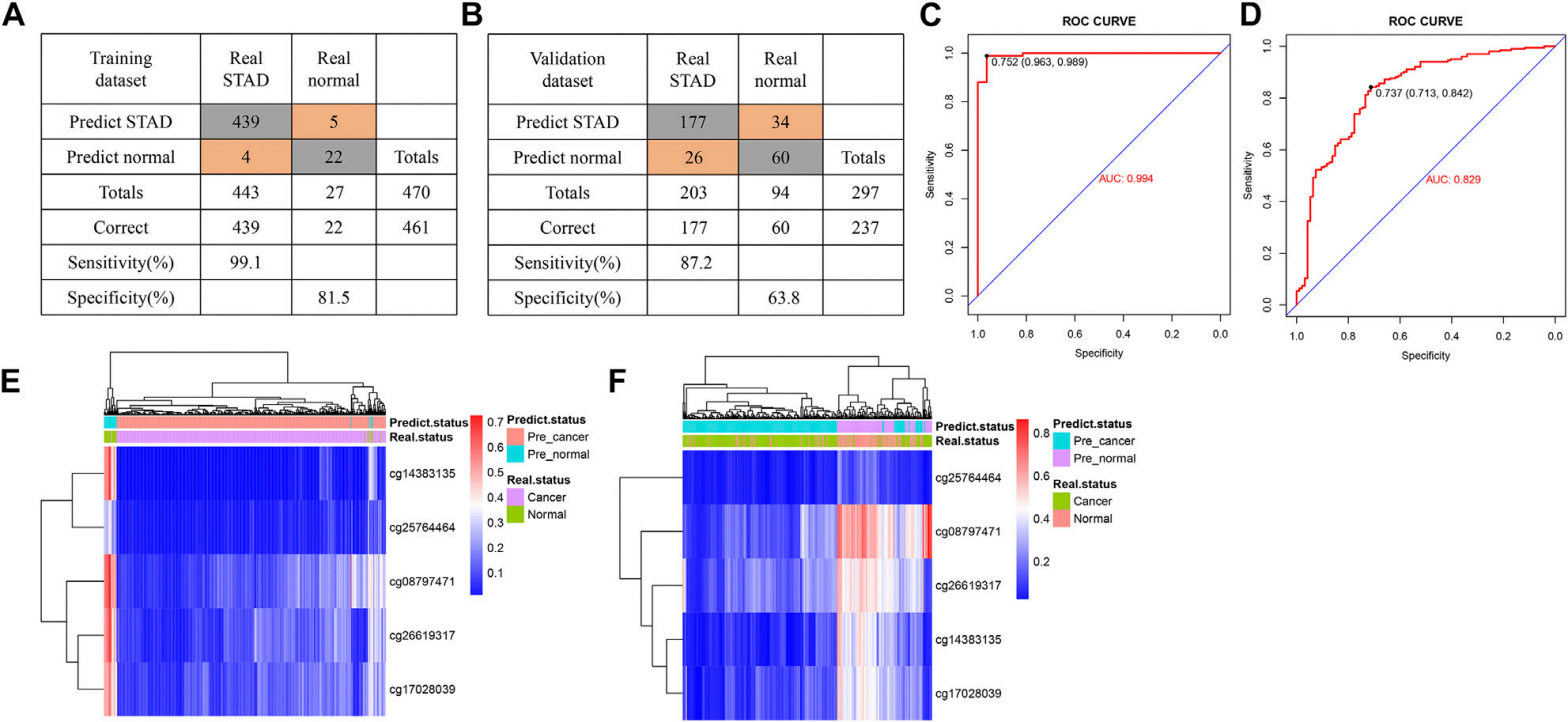
Construction of a diagnostic model of DNA methylation (DNAm) in gastric cancer. **(A,B)** Confusion tables of binary results of the diagnostic prediction model in the training **(A)** and validation datasets **(B)**. **(C,D)** Receiving operating characteristics curve analysis of the diagnostic prediction model with DNAm markers in the training **(C)** and validation datasets **(D)**. **(E,F)** Unsupervised hierarchical clustering of five DNAm markers selected for use in the diagnostic prediction model in the training **(E)** and validation data sets **(F)**.

Using multivariate logistic regression analysis, we established a GC diagnosis prediction model with the five selected DNAm markers (Table 2). Applying the model to the training dataset yielded a sensitivity of 99.1% and specificity of 81.5% samples (Figure 3A) and a sensitivity of 87.2% and specificity of 63.8% in the validation dataset (Figure 3B). We also demonstrated this model could differentiate GC from normal samples both in the training dataset (AUC = 0.994) and the validation dataset (AUC = 0.829) (Figures 3C,D). Unsupervised hierarchical clustering of these five markers distinguished GC from normal samples with high specificity and sensitivity (Figures 3E,F). These results indicated that the DNAm diagnostic model could be a significant tool for distinguishing GC from normal samples.

**TABLE 2:** Characteristics of five methylation markers and their coefficients in GC diagnosis.

Markers	Ref.Gene	Coefficients	SE	z.value	P.value
		12.209	3.242	3.766	<0.001
cg14383135	NPAS2	−2.609	7.309	−0.357	0.721
cg08797471	DAPK1	−19.390	5.950	−3.259	0.001
cg26619317	CNN3	−2.969	7.454	−0.398	0.690
cg17028039	FGFR2	−6.982	9.783	−0.714	0.475
cg25764464	PLEKHA5	−2.097	6.914	−0.303	0.762

SE: standard errors of coe-cients; z value: Wald z-statistic value.

### Correlation Between DNAm Diagnostic Model and Clinical Indicators

**FIGURE 4:**
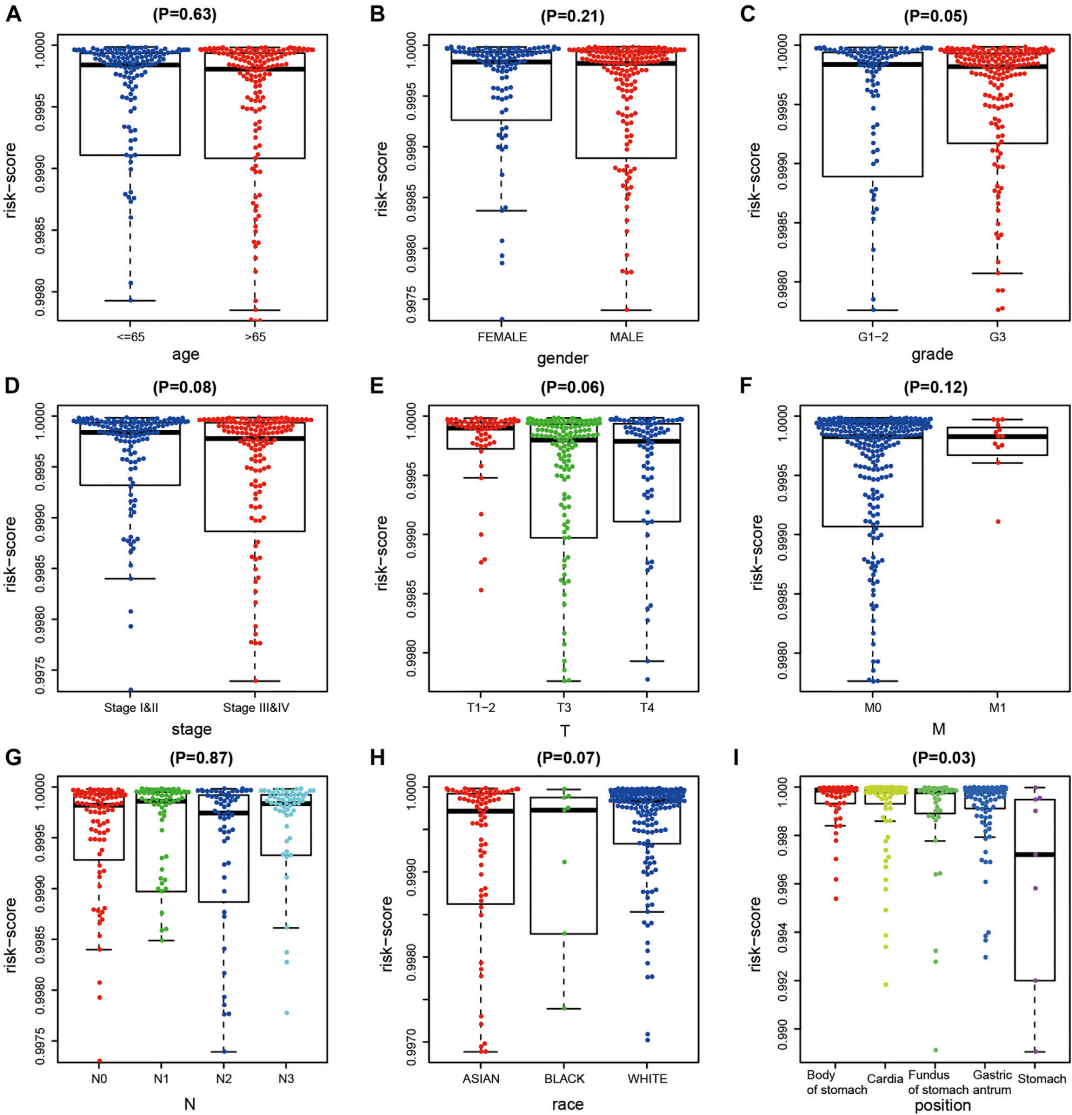
Correlation analysis of DNA methylation (DNAm) diagnostic model and clinical indicators in gastric cancer (GC). **(A–I)** Correlation analysis between diagnostic risk score and age, gener, tumor grade, T, M, N stage, race, and tumor site of gastric cancer.

After excluding samples with missing clinical data, we analyzed correlations between the diagnostic risk score and the clinical characteristics of 323 patients obtained from TCGA dataset. The results indicated that diagnostic risk score was significantly correlated with grade and tumor location in patients with GC, but not with age, gender, stage, extent of the tumor (T), presence of metastasis (M), extent of spread to the lymph nodes (N), or race of the patient (Figures 4A–I).

### Prognostic Model Based on Differential Methylated Sites

**FIGURE 5:**
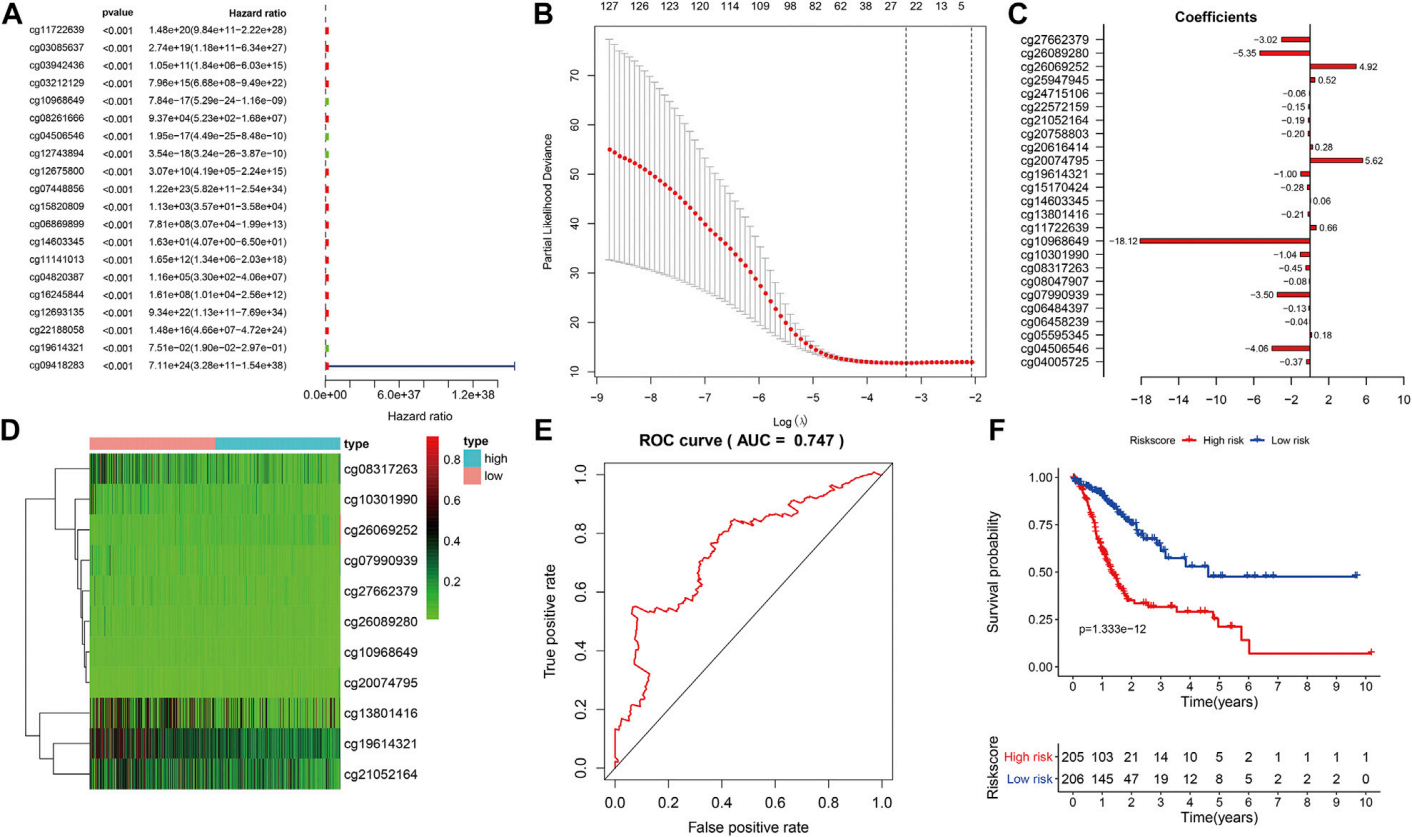
Prognostic model based on differential methylation sites (DMSs). **(A)** Random forest plot of the top DMS with the most significant differences through univariate Cox proportional hazard regression analysis. **(B)** Results of LASSO regression analysis and 10,000 simulations. **(C)** Corresponding coefficient values of each DMS in LASSO regression analysis. **(D)** DNAm heatmap of DMSs in the low-risk and high-risk groups with increasing prognostic risk score value. **(E)** Receiver operating characteristics curve analysis of the prognostic model. **(F)** Kaplan–Meier survival analysis of DMSs related to prognostic model, abscissa for survival time, ordinate for survival rate, blue curve for low-risk patients, red curve for high-risk patients. The number of high-risk and low-risk patients at each time point are located on the bottom axis of the graph.

We combined the DNAm values of the DMSs in GC samples with the survival data of the corresponding patients, using *p* < 0.01 as the threshold standard to perform univariate Cox proportional hazard regression analysis. We found that 137 DMSs significantly affected the survival of patients with GC, among which the top 20 DNAm sites with the most significant differences are shown (Figure 5A). We used LASSO regression analysis to remove redundant DNAm sites, performed 10,000 simulations, removed overlaps through cross validation, and finally obtained 25 prognostic-related DMSs (Figures 5B,C). We constructed a prognostic risk score formula for each patient based on these 25 prognosis-related DMSs (Table 3). The DNAm heatmap demonstrated the DMSs in the low-risk and high-risk groups based on the prognostic (Figure 5D). The corresponding ROC curve analysis demonstrated that the area under the curve (AUC) value of the constructed prognostic model was 0.747, which indicated the predictive power of the prognostic model based on the expression of DMSs in GC (Figure 5E). Further, the Kaplan–Meier curves suggested that the survival rate of patients in the high-risk group was significantly lower than that in the low-risk group (Figure 5F).

**TABLE 3:** Characteristics of eleven methylation markers and their coefficients in GC prognosis prediction.

Markers	Ref.Gene	Coefficients	HR	Cl	SE	z.value	P.value
cg07990939	CEP290	−8.908	1.35E-04	2.05e-07–8.94e-02	3.313	−2.689	7.17E-03
cg08317263	CCDC69	−1.739	1.76E-01	3.38e-02–9.13e-01	0.841	−2.068	3.86E-02
cg10301990	UBXN8	−4.088	1.68E-02	1.99e-04–1.41e+00	2.263	−1.807	7.08E-02
cg10968649	KDM4A	−20.267	1.58E-09	1.73e-17–1.44e-01	9.352	−2.167	3.02E-02
cg13801416	AKR1B1	−1.009	3.65E-01	1.65e-01–8.05e-01	0.404	−2.496	1.26E-02
cg19614321	RASSF2	−1.779	1.69E-01	3.55e-02–8.02e-01	0.795	−2.237	2.53E-02
cg20074795	KDELR3	12.778	3.54E+05	1.54e+01–8.15e+09	5.124	2.494	1.26E-02
cg21052164	CHRNB2	−0.941	3.90E-01	1.22e-01–1.24e+00	0.592	−1.59	1.12E-01
cg26069252	EGR1	7.734	2.29E+03	1.69e+02–3.08e+04	1.327	5.826	5.67E-09
cg26089280	ARMC9	−8.569	1.90E-04	1.60e-09–2.25e+01	5.96	−1.438	1.51E-01
cg27662379	RPN1	−7.672	4.66E-04	1.65e-07–1.31e+00	4.053	−1.893	5.84E-02

HR: Hazard Ratio; CI: 95.0% confidence interval; SE: standard errors of coe-cients; z value: Wald z-statistic value.

### Analysis of Independent Prognostic Factors in the Prognostic Risk Model

**FIGURE 6:**
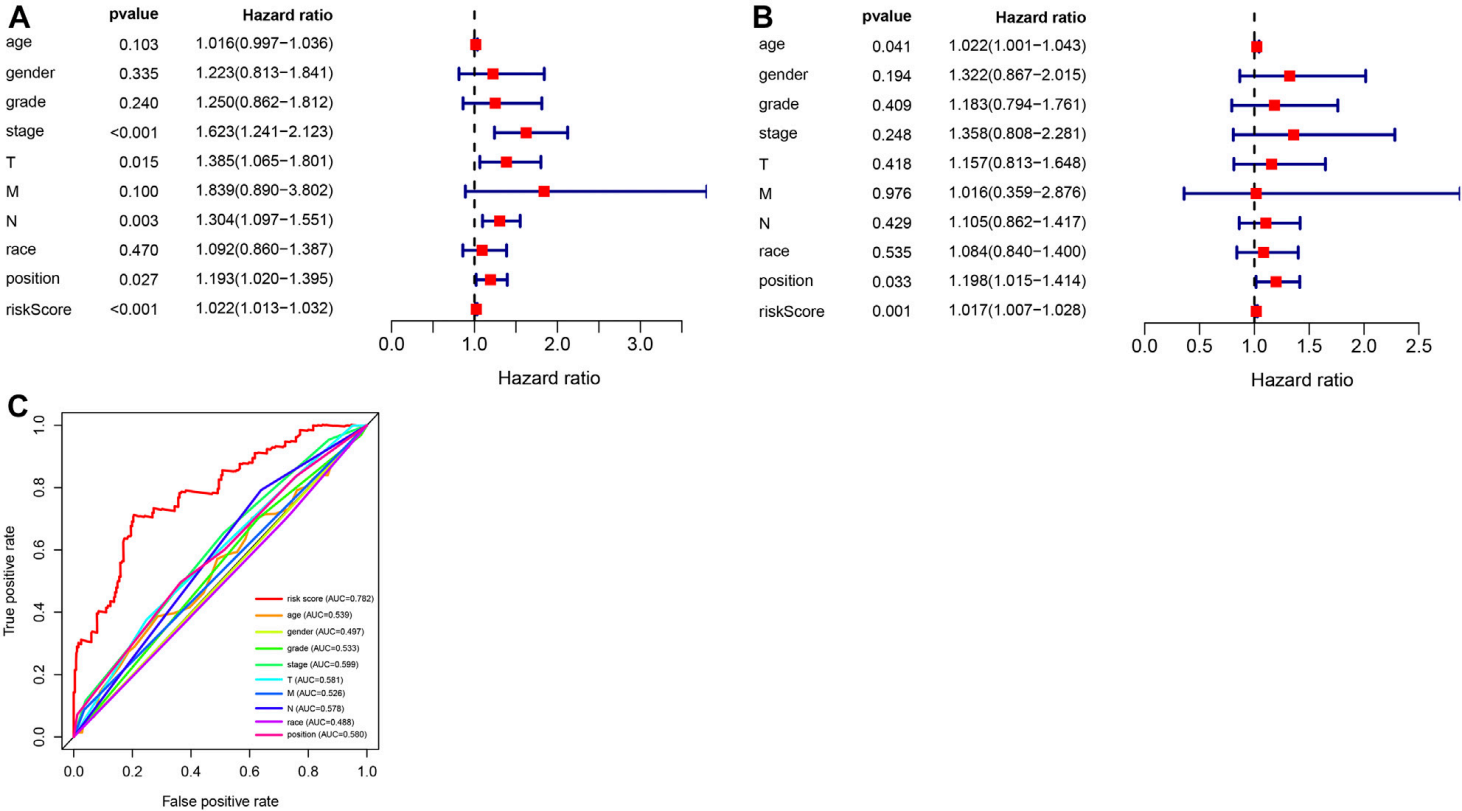
Analysis of independent prognostic factors in the prognostic risk model. **(A)** Random forest plot of univariate independent prognostic analysis; the left side indicates clinical characteristics of gastric cancer (GC), the middle is the *p*-value. The hazard ratio indicates the risk rate with hazard ratio >1 indicating high-risk clinical features, and hazard ratio <1 indicating low-risk clinical features. **(B)** Random forest plot of multivariate independent prognostic analysis; the left side represents clinical characteristics of GC, the middle is the *p*-value. The hazard ratio represents the risk rate with hazard ratio >1 indicating high-risk clinical features, and hazard ratio <1 indicating low-risk clinical features. **(C)** Receiver operating characteristics curve analysis of the prognostic model constructed with eleven differential methylation sites (DMSs) in GC.

To further evaluate the prognostic model and the impact of different clinical characteristics of patients with GC on prognosis and survival, we obtained the corresponding age, gender, phenotype, and clinical information for 315 patients with GC from TCGA dataset. We performed univariate and multivariate independent prognostic analyses (Figures 6A,B), revealing that the prognostic risk score value and tumor site were significant high-risk factors and were significantly correlated with the survival status of patients with GC (*p* < 0.05). The corresponding ROC curve analysis demonstrated that the constructed prognostic model had the largest AUC value of 0.782, which also indicated the predictive power of the prognostic model based on DMSs in GC (Figure 6C).

### Functional Analysis of Prognostic Risk Score

**FIGURE 7:**
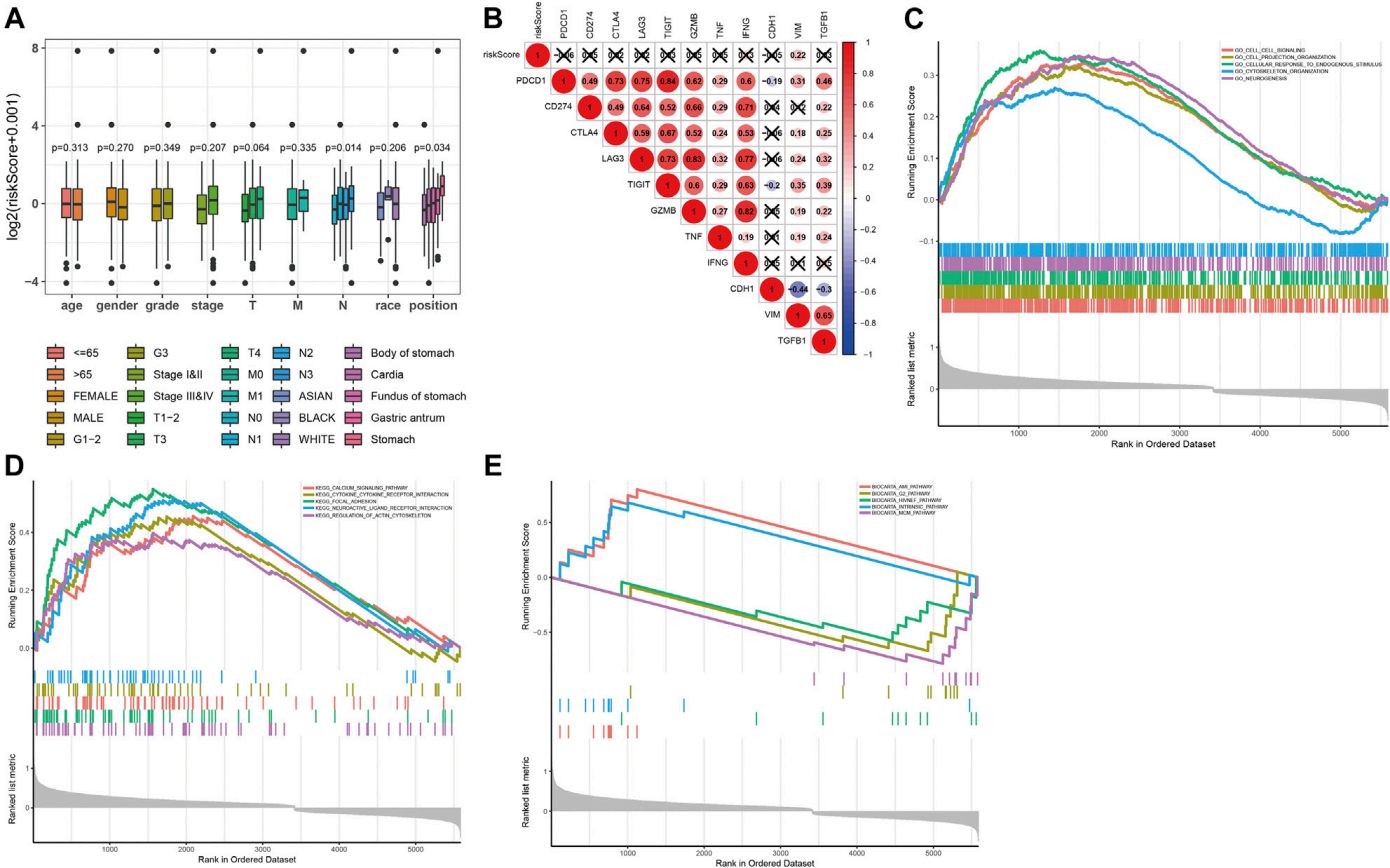
Functional analysis of prognostic risk score. **(A)** Correlation analysis between prognostic risk score and age, gender, tumor grade, N stage, T stage, race, and tumor location of the patient in the prognostic risk model. **(B)** Correlation analysis between prognostic risk score and expression levels of regulatory, cytotoxic, and epithelial–mesenchymal transition (EMT) factors of immune checkpoint sites. **(C–E)** The results of gene set enrichment analysis on the potential mechanism of c5 (c5.bp.v7.1.entrez.gmt) and c2 (c2.cp.kegg.v7.1.entrez.gmt, c2.cp.biocarta.v7.1.entrez.gmt) in the molecular signatures database.

To evaluate the clinical application and important functions of the DNAm prognostic model in GC, we calculated the prognostic risk score of patients with GC from TCGA dataset and then analyzed correlations with patient clinical characteristics. The prognostic risk score was significantly correlated with extent of spread to the lymph nodes (N) and tumor site in patients with GC but not significantly correlated with other clinical features (Figure 7A). We also analyzed correlations between prognostic risk score and expression levels of regulatory, cytotoxic, and EMT factors of immune checkpoint sites. The results indicated that prognostic risk score was significantly positively correlated with VIM, which was significantly positively correlated with PDCD1, CTLA4, LAG3, TIGIT, GZMB, and TNF and significantly negatively correlated with CDH1 (Figure 7B). We screened 6,172 significant differentially expressed genes in the high-risk group samples. GSEA on the potential mechanism of c2 (c2.cp.kegg.v7.1.entrez.gmt, c2.cp.biocarta.v7.1.entrez.gmt) and c5 (c5.bp.v7.1.entrez.gmt) in the MSigDB (Figures 7C–E) revealed that highly expressed genes in the high-risk group were significantly enriched in multiple biological processes, such as the “calcium signaling pathway,” “cytokine receptor interaction,” “focal adhesion,” “neuroactive ligand receptor interaction,” and “regulation of actin cytoskeleton,” indicating that these pathways may play important roles in the development of GC.

### Analysis of the Correlation Between Eleven Prognostic-Related DMG and TP53 Mutations

**FIGURE 8:**
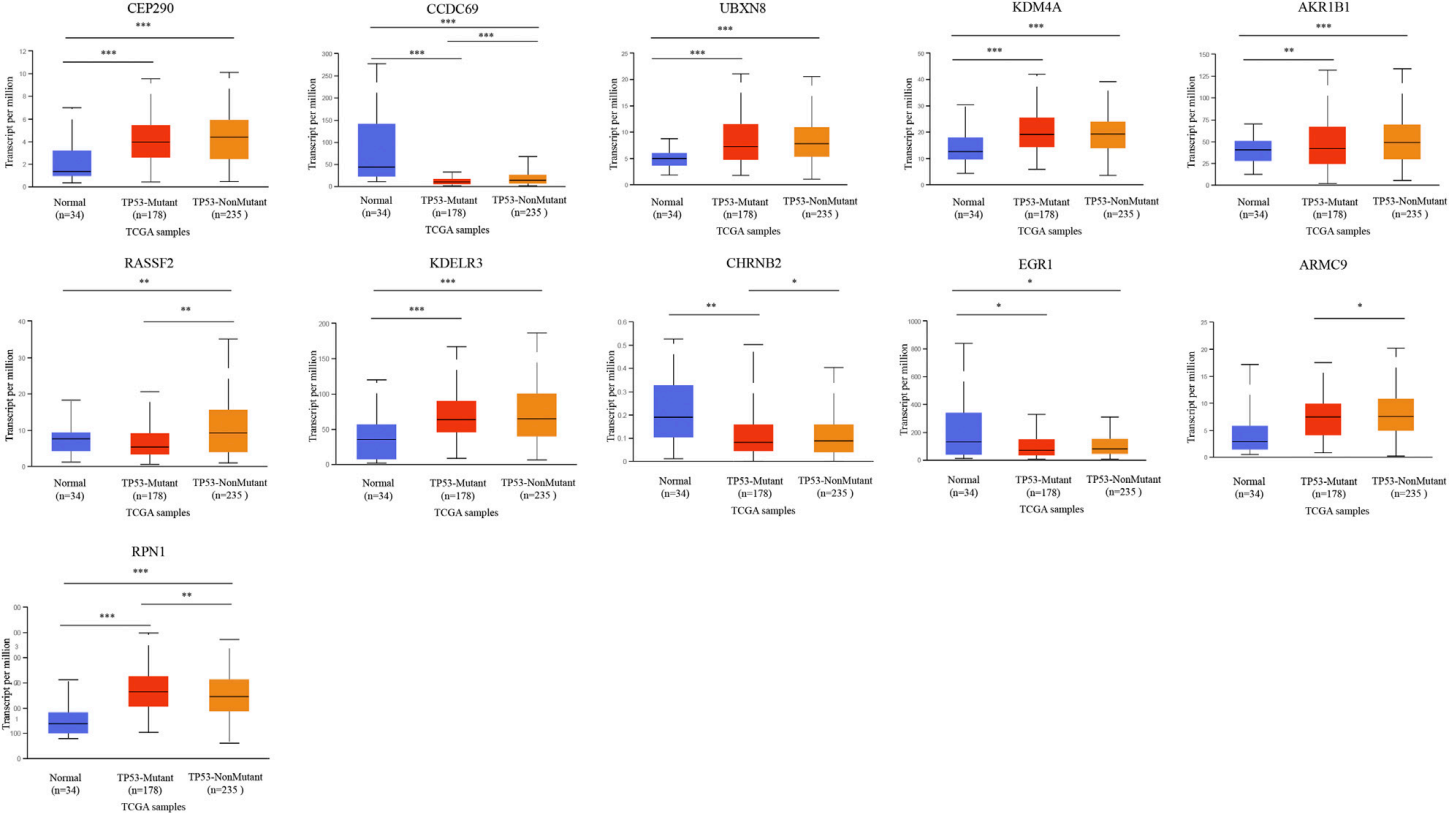
The relationship between 11-DMG mRNA expression levels and TP53 mutation in gastric cancer (GC) (mutation: red, non-mutation: orange, and normal gastric tissues: blue) (UALCAN) (*p < 0.05, **p < 0.01, ***p < 0.001).

We further analyzed the relationship between DMG mRNA expression levels and TP53 mutation status in patients with gastric cancer using the UALCAN data mining website. In the correlation analysis of TP53 mutation status, it is worth noting that the expression of CHRNB2 decreased significantly only in the TP53 mutation group of gastric cancer patients. CCDC69, RASSF2, CHRNB2, ARMC9, and RPN1 were significantly different in the TP53 mutation group and TP53 non-mutation group of gastric cancer patients (Figure 8).

### Mutation and Prognostic Analysis of Eleven Prognostic-Related DMG

**FIGURE 9:**
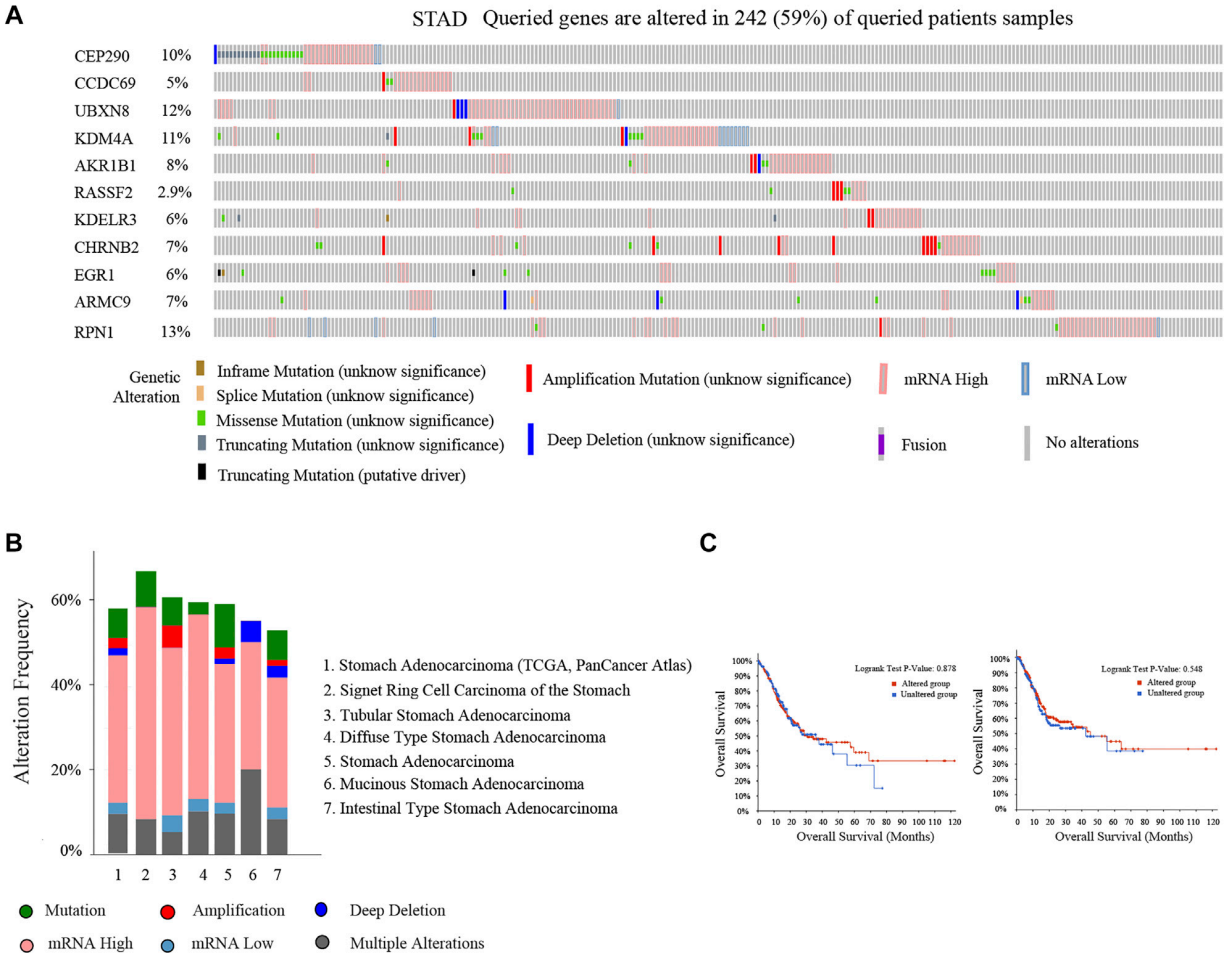
Mutation of 11-DMG in gastric cancer (GC) patients (cBioportal). **(A)** A visual summary of 11-DMG mutation frequency. **(B)** Summary of mutation frequency of 11-DMG in gastric cancer patients. **(C)** Kaplan-Meier plotter was used to compare the relationship between gene mutation (red) and gene non-mutation (blue) of 11-DMG mutation with OS and PFS (*p* < 0.05 statistical significance).

**FIGURE 10:**
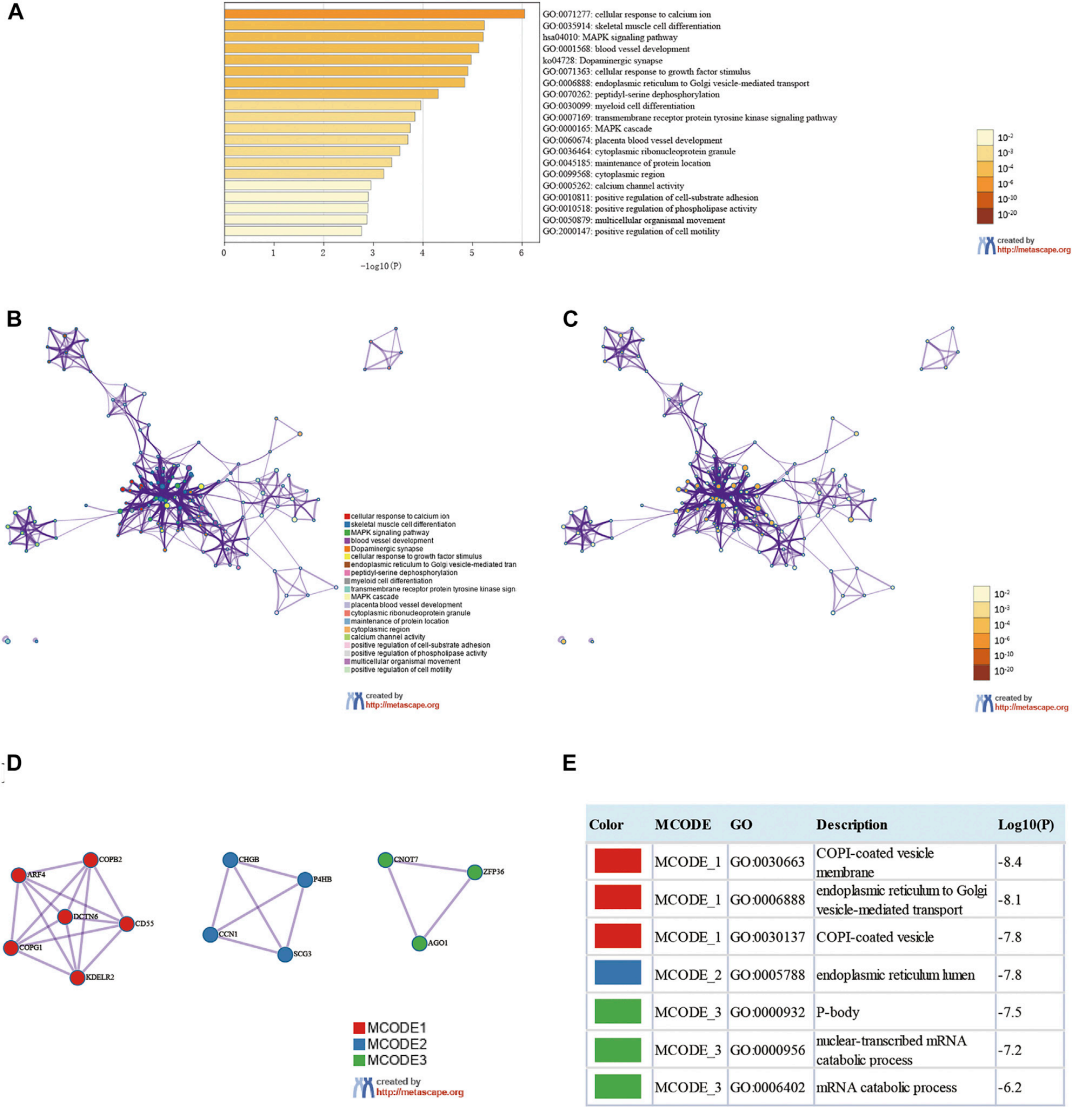
Enrichment analysis of genes related to 11-DMG mutation in gastric cancer (GC) (Metascape). **(A)** Heat maps of Go and KEGG enrichment analysis of 108 adjacent genes related to 11-DMG mutation were stained with *p*-value. **(B)** Term-enriched network: colored by cluster ID, where nodes sharing the same cluster ID are usually close to each other, **(C)** colored by *p*-value, terms containing more genes tend to have more significant *p*-values. **(D)** For the MCODE components identified in the protein-protein interaction network, **(E)** the three best score items divided by *p*-value are used as the functional description of the corresponding components, which are represented by the grid diagram.

We analyzed eleven prognostic-related DMG mutations and their relationship with OS and PFS in gastric cancer patients using the cBioportal website. Among 412 patients with gastric cancer, 242 had gene mutations, with a mutation rate of 59%. The mutation rates of CEP290, CCDC69, UBXN8, KDM4A, AKR1B1, RASSF2, KDELR3, CHRNB2, EGR1, ARMC9, RPN1 were 10, 5, 12, 11, 8, 2.9, 6, 7, 6, 7, and 13%, respectively. We observed that the mutation rates of CEP290, UBXN8, KDM4A, and RPN1 were more than 10% (10, 12, 11, 13%) (Figure 9A). In addition, high mRNA expression was an important factor leading to high mutation frequency in gastric cancer (Figure 9B). However, Kaplan-Meier plotter and log-rank test analysis showed that SMYD family mutations had no significant correlation with OS and PFS in patients with gastric cancer (OS: *p* value = 0.887, PFS: *p* value = 0.548) (Figure 9C). Next, we used the cBioportal to search for genes that were significantly related to gastric cancer and DMG mutations (the top 10, respectively). After deduplication, a total of 108 genes were obtained, ZDHHC17, ARID4A, ATRX, ARID4B, UPF2, ZNF37BP, CEP162, MDM4, CCDC66, PHIP, ASB2, PRKCB, GYPC, SLC9A9, RASGRP2, JAM2, FNBP1, MAP3K3, PLEKHO, GTF2E2, MAK16, CNOT7, PPP2CB, CCDC25, DCTN6, INTS10, PPP2R2A, LEPROTL1, ELP3, AGO1, PTPRF, COMMD6, NCOA2, COPS9, MRPL53, POLR3A, UHMK1, CSNK1G1, AIDA, ADAP2, NRROS, HVCN1, LY86, TM6SF1, TRPV2, MAP7, CSF1R, CHST11, TNFAIP8L2, FLI1, ARHGEF6, ZEB2, RCSD1, MEF2C, FMNL3, ARHGAP31, CYRIA, SYNE1, GIMAP8, CREB3L1, ARF4, AGR2, KCNK1, SEC13, BACE2, CD55, KDELR2, S100P, BSN, RUNDC3A, CHGB, SCG3, AP3B2, SYP, CACNA2D2, SEZ6, CELF3, GNG4, FOS, FOSB, ZFP36, DUSP1, CSRNP1, NR4A1, JUNB, EGR3, CCN1, ATF3, COL8A1, MAP1A, PKD2, EDNRA, AEBP1, TIMP2, SYDE1, KANK2, SCARF2, DDR2, SEC61A1, COPG1, SRPRB, TFG, P4HB, COPB2, UMPS, TMEM39A, RUVBL1 and PDIA5, respectively. The 108 genes significantly related to 11-DMG mutation obtained from the cBioportal were used through the Meatascape website to perform GO and KEGG enrichment analysis (Figures 10A–C). GO enrichment was divided into three functional groups: biological processes (15 items), molecular functions (1 item), and cellular components (2 items), and KEGG functional group (2 items). We found that these genes were mainly involved in cellular response to calcium, skeletal muscle cell differentiation, blood vessel development, cellular response to growth factor stimulus, endoplasmic reticulum to Golgi vesicle-mediated transport, peptidyl-serine dephosphorylation, myeloid cell differentiation, transmembrane receptor protein tyrosine kinase signaling pathway, MAPK cascade, placenta blood vessel development, maintenance of protein location, positive regulation of cell-substrate adhesion, positive regulation of phospholipase activity, multicellular organismal movement, positive regulation of cell motility. The molecular function of these genes mainly played a role in the activity of calcium channels. The cellular components involved in these genes were cytoplasmic ribonucleoprotein granules and cytoplasmic regions (Table 4). In addition, in order to better understand the relationship between DMG mutation-related genes and GC, we conducted protein interaction network analysis. After pathway and process enrichment analysis for each MCODE component, it was found that the main component of the cell involved was the endoplasmic reticulum lumen, and the biological function was mainly related to COPI-coated vesicle membrane, endoplasmic reticulum to Golgi vesicle-mediated transport, COPI-coated vesicle, P-body, nuclear-transcribed mRNA catabolic process, mRNA catabolic process (Figures 10D–E).

**TABLE 4:** The GO and KEGG function enrichment analysis of genes related to 11-DMG mutation in GC.

GO	Category	Description	Count	%	Log_10_(P)	Log_10_(q)
GO:0071277	GO Biological Processes	cellular response to calcium ion	6	5.61	-6.05	-1.71
GO:0035914	GO Biological Processes	skeletal muscle cell differentiation	5	4.67	-5.24	-1.69
hsa04010	KEGG Pathway	MAPK signaling pathway	8	7.48	-5.21	-1.69
GO:0001568	GO Biological Processes	blood vessel development	13	12.15	-5.13	-1.69
ko04728	KEGG Pathway	Dopaminergic synapse	6	5.61	-4.97	-1.69
GO:0071363	GO Biological Processes	cellular response to growth factor stimulus	12	11.21	-4.90	-1.69
GO:0006888	GO Biological Processes	endoplasmic reticulum to Golgi vesicle-mediated transport	6	5.61	-4.84	-1.68
GO:0070262	GO Biological Processes	peptidyl-serine dephosphorylation	3	2.80	-4.31	-1.24
GO:0030099	GO Biological Processes	myeloid cell differentiation	8	7.48	-3.96	-1.01
GO:0007169	GO Biological Processes	transmembrane receptor protein tyrosine kinase signaling pathway	10	9.35	-3.84	-0.92
GO:0000165	GO Biological Processes	MAPK cascade	11	10.28	-3.74	-0.89
GO:0060674	GO Biological Processes	placenta blood vessel development	3	2.80	-3.70	-0.86
GO:0036464	GO Cellular Components	cytoplasmic ribonucleoprotein granule	6	5.61	-3.54	-0.71
GO:0045185	GO Biological Processes	maintenance of protein location	4	3.74	-3.37	-0.62
GO:0099568	GO Cellular Components	cytoplasmic region	6	5.61	-3.21	-0.53
GO:0005262	GO Molecular Functions	calcium channel activity	4	3.74	-2.95	-0.38
GO:0010811	GO Biological Processes	positive regulation of cell-substrate adhesion	4	3.74	-2.90	-0.36
GO:0010518	GO Biological Processes	positive regulation of phospholipase activity	3	2.80	-2.89	-0.36
GO:0050879	GO Biological Processes	multicellular organismal movement	3	2.80	-2.87	-0.36
GO:2000147	GO Biological Processes	positive regulation of cell motility	8	7.48	-2.76	-0.29

It includes the first 20 clusters and their representative enrichment terms (one for each cluster). “Count” is the number of genes in the provided list that have membership in the given ontology term. “%” is the percentage of all genes provided found in a given ontology term (only input genes with at least one ontology term annotation are included in the calculation). “Log10(P)” is the *p* value based on Log10. “Log10(q)” is a multi-test adjusted *p* value based on Log10.

### Construction of Multi-Factor Regulatory Network of Key Genes

**FIGURE 11:**
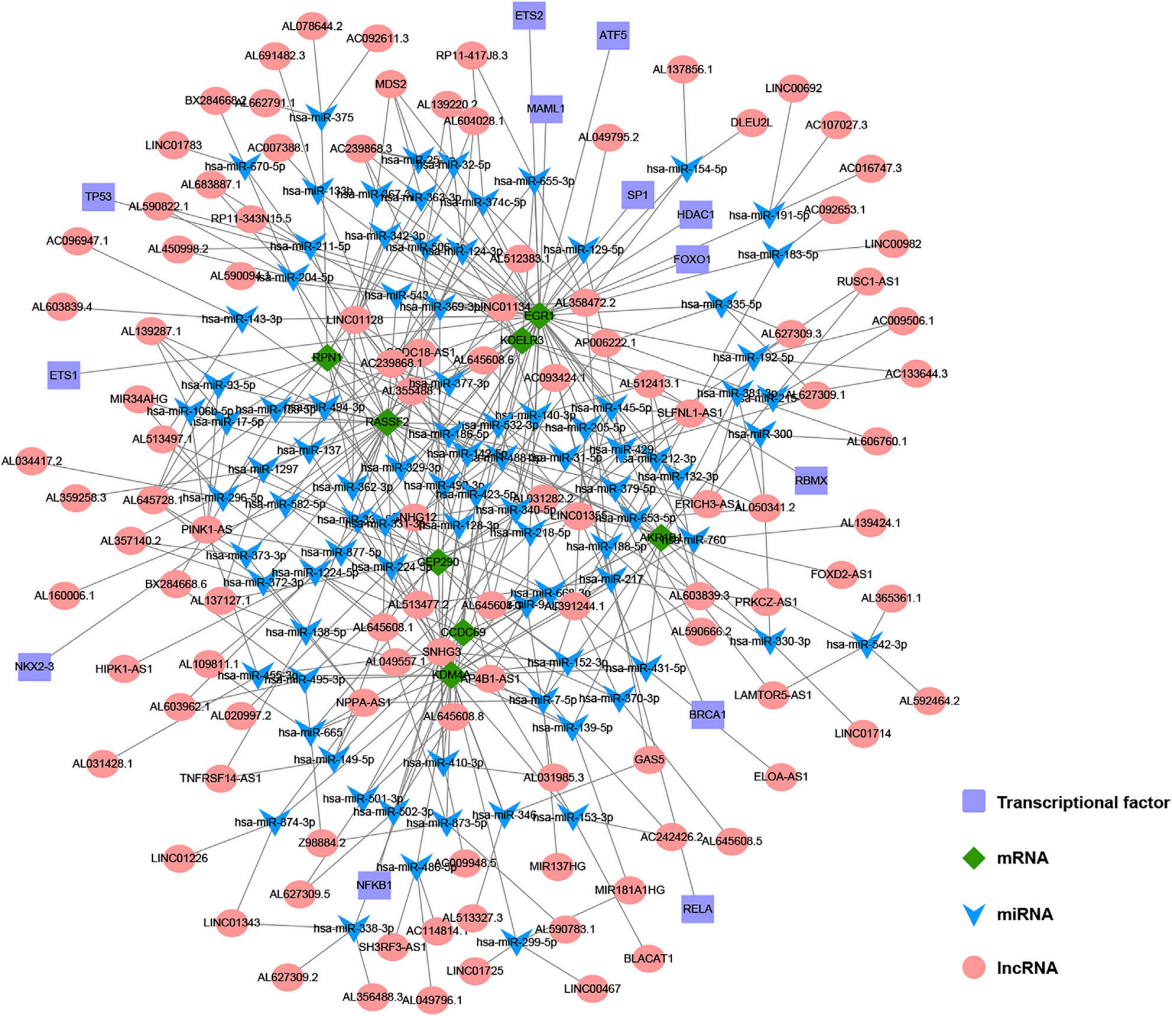
The construction of multi-factor regulatory network of key genes in gastric cancer (GC). Diamond represents mRNA, V-shape represents miRNA, circle represents lncRNA, and rectangle represents TF.

Using databases such as Starbase, TargetScan and other databases to predict the miRNAs upstream regulated of 11 key genes, and intersect the prediction results, a total of 90 reliable miRNAs capable of regulating 11 mRNAs were obtained. By predicting the upstream of reliable miRNA regulated lncRNAs through the Starbase database to, a total of 2,469 lncRNAs were obtained, and the most reliable first three lncRNAs were selected for each miRNA, and finally 270 credible lncRNAs were obtained. The TRRUST database predicted transcription factors that can regulate 11 key genes, and 13 TFs were obtained. Finally, the regulatory network between mRNA, miRNA, lncRNA and TF was constructed (Figure 11).

## Discussion

Although tumor markers for different types of cancers have been rapidly discovered in recent years, there remains a lack of specific and sensitive tumor markers for the management of GC. With the development and deeper understanding of epigenetics, abnormal DNAm has become the most extensively studied epigenetic mechanism in GC research, and the relationship between DNAm and tumors has become a research hotspot. The mechanism whereby DNAm promotes cancer may be related to activation or inhibition of certain signaling pathways, and DNAm is thus recognized as a potential tumor marker (Rashid and Issa, 2004). However, the performance of a single DNAm site in predicting the prognosis of GC is unreliable. A large prospective trial with 7,941 patients with colorectal cancer was conducted to evaluate the accuracy of screening circulating DNAm by detecting the methylation level of SEPT9. The results revealed a specificity of 91.5% but a sensitivity of only 48.2% (Church et al., 2014). Some studies have shown that the prediction accuracy of GC models is improved by combining multiple tumor markers (Li et al., 2020a; Bai et al., 2020). This is because multiple markers can take advantage of the complementary effects of genetic information and effectively eliminate redundant genes through machine learning algorithms. As a result, we developed a GC diagnostic model with a 5-DMS signature and a GC prognostic model with an 11-DMS signature. Through clinical correlation analysis of the diagnostic models, independent prognostic factors analysis of prognostic models and enrichment analysis of the high-risk prognostic risk score group, our study provides potential targets and related mechanisms for clinical diagnosis and treatment of GC.

The accuracy of a DNAm diagnostic model has been confirmed for liver cancer (Luo et al., 2020). In the current study, we developed a 5-DMS (NPAS2, DAPK1, CNN3, FGFR2, PLEKHA5) signature diagnostic model and calculated GC diagnostic risk scores to accurately distinguish GC from normal tissues. The predicted results were highly consistent with the actual results, indicating the model’s potential for wide application. In addition, unsupervised hierarchical clustering analysis demonstrated high specificity and sensitivity. In subsequent analysis, the diagnostic risk score was significantly correlated with grade and tumor site in patients with GC. Since the disease state of gastric cancer patients is often manifested in clinical characteristics, the correlation analysis between the risk score calculated by this diagnostic model and the clinical characteristics can further understand the quality of our model and assess the clinical status of GC patients, which is of great significance. In clinical practice, the gold standard for GC diagnosis is pathological results, but the diagnostic model still has high clinical value. At the same time, this model and pathology are used for diagnosis. If the two diagnostic results are consistent, it is more convincing. Generally, pathological diagnosis is the main method, and model diagnosis is the auxiliary method. In addition, the model can assist in the diagnosis and classification of patients with difficult pathological diagnosis, and can also be used for the detection of tumor residual, recurrence and metastasis for subsequent accurate and personalized treatment.

The prognostic model constructed in the current study employed an 11-DMS (CEP290, CCDC69, UBXN8, KDM4A, AKR1B1, RASSF2, KDELR3, CHRNB2, EGR1, ARMC9, and RPN1) signature. In this model, prognostic risk score effectively distinguished patients with GC into high-risk and low-risk groups. Kaplan–Meier curves also confirmed that the survival rate of patients in the high-risk group was significantly lower than that in the low-risk group. By univariate and multivariate Cox analyses, prognostic risk score was proven to be an independent prognostic risk factor for GC. Compared with other clinical factors (age, gender, tumor grade, clinical stage, T, N, and M stage, race, tumor location), prognostic risk score had higher predictive potential, which indicated the reliability of the model for predicting the prognosis of patients with GC. Although TNM stage is still the gold standard for the classification and prognosis of GC patients, from the perspective of data analysis, this prognostic model can better reflect the prognosis of gastric cancer patients than TNM stage. With the continuous expansion of subsequent data, the constructed prognostic model will with higher stability and accuracy, it is not impossible to replace TNM stage. In clinical practice, we often encounter GC patients with the same TNM stage and other clinical characteristics, but their prognosis is quite different, and the subsequent treatment plans given are not completely the same. For this situation, we can apply this prognostic model to classify and predict the prognosis, so that doctors can summarize the treatment plans of patients in the high-risk group and the low-risk group, and provide corresponding treatment plans. Therefore, this prognostic model has great potential value in the prognosis judgment and treatment of GC patients, which is helpful for accurate and personalized treatment in the clinical environment.

Among the eleven DMGs in the prognostic model, five DMGs (KDM4A, AKR1B1, RASSF2, CHRNB2, and EGR1) are known to be closely related to the occurrence and development of GC. The protein encoded by the KDM4A gene acts as a trimethylation-specific demethylase, which can specifically demethylate the “Lys-9” and “Lys-36” residues of histone H3, thereby playing a central role in coding for histones (Bavetsias et al., 2016). This protein can also control the growth and invasion of GC cells by inhibiting the KDM4A/YAP1 pathway (Chen et al., 2019). The AKR1B1 gene encodes a member of the aldose/keto reductase superfamily, which is composed of more than 40 known enzymes and proteins. The related pathways include acetone degradation I (conversion to methylglyoxal) and glycerolipid metabolism (Sivenius et al., 2004; Wolford et al., 2006). AKR1B1 plays an important role in the occurrence and development of GC, which had a certain reference value for the prognosis of patients with GC (Li et al., 2020b). The protein encoded by the RASSF2 gene has been found to be a potential tumor suppressor and can act as a KRAS-specific effector protein. It may promote apoptosis and cell cycle arrest, stabilizing STK3/MST2 by protecting it from proteasome degradation (Cooper et al., 2009). Meta-analysis has shown that RASSF2 is significantly more methylated in GC, which can predict the risk of GC (Zhou et al., 2019c). Neuronal acetylcholine receptors are homo- or heteropentameric complexes composed of homologous α and β subunits, of which the CHRNB2 gene encodes one of several β subunits. The related pathways include nicotine addiction and chemical synaptic transmission (Chen et al., 2009). CHRNB2 and TP53 may also play a role in *Helicobacter* pylori-associated GC, but the specific mechanism is unknown (Hu et al., 2018). The protein encoded by the EGR1 gene belongs to the EGR family of C2H2-type zinc-finger proteins and is a transcriptional regulator (Hu et al., 2010). Its functions are diverse and can regulate the transcription of many target genes, thus, playing an important role in regulating the response to growth factors, DNA damage, and ischemia. Its role in regulating cell survival, proliferation, and cell death cannot be ignored. EGR1 protein can directly bind to the HNF1A-AS1 promoter region and activate its transcription to promote the GC cell cycle (Liu et al., 2018). The relationship between the remaining six DMGs and GC is unknown. Further exploration of the potential functions and mechanisms of these DMGs may deepen our understanding of GC development and provide potential tumor markers.

Regulatory, cytotoxic, and EMT factors are significantly associated with the occurrence, development, and immunity of tumor (Zhou et al., 2019b), and their analysis can further explore potentially important biological phenotypes. Correlation analysis with these three factors revealed that prognostic risk score was significantly positively correlated with VIM. This gene encodes a type III intermediate filament protein responsible for maintaining cell shape and cytoplasm integrity and stabilizing cytoskeletal interactions. VIM protein is involved in neurogenesis, cholesterol transport, and functions as an organizer of a number of other critical proteins involved in cell attachment, migration, and signaling. EMT is widespread in malignant tumor cells, of which VIM is a marker gene. The higher the risk score, the more likely EMT will occur. We performed GSEA to clarify the potential mechanisms involved in GC that were identified in the high-risk score group. The differentially expressed genes were mainly distributed in five pathways: “calculation signaling pathway,” “cytokine receptor interaction,” “focal assignment,” “neural ligand receptor interaction,” and “regulation of actin cytoskeleton.” This indicates that the above pathways may be related to the origin of GC, which concurs with the results of previously published research (Liu et al., 2016; Zhu et al., 2017; Xu et al., 2019; Zhou et al., 2020).

In order to understand the correlation between 11-DMG and TP53 mutation, we analyzed their correlation on the data website through UALCAN. In the analysis, we found for the first time that the expression of CHRNB2 was significantly reduced only in the TP53 mutation group of gastric cancer patients, and the mutation of tumor suppressor gene TP53 may be involved in the regulation of mRNA expression in CCDC69, RASSF2, CHRNB2, ARMC9, and RPN1(Sartorio and Morabito, 1988; Hu et al., 2018; Wang et al., 2020). In the analysis of 11-DMG mutation and prognosis, we found that CEP290, UBXN8, KDM4A, RPN1 had high frequency mutations. The genes related to their mutations are mainly related to pathways such as COPI-coated vesicle membrane, endoplasmic reticulum to Golgi vesicle-mediated transport, COPI-coated vesicle, P-body, nuclear-transcribed mRNA catabolic process, mRNA catabolic process.

To the best of our knowledge, the 5-DMS diagnostic and 11-DMS prognostic models of GC have not been previously reported. The models were verified by external datasets and demonstrated good generalization ability, which can facilitate clinical treatment decision-making. The DMSs selected in this study are relatively novel, and subsequent research on these DMSs will be of great significance. However, this study also has some shortcomings. The small normal sample size may lead to some bias in the results. Other omics fields, such as genome, transcriptome, proteome, and metabolome, have shown respective advantages in GC diagnostic and prognostic models (Li et al., 2010; Chan et al., 2016; Deng et al., 2018; Zhang et al., 2018; Shen et al., 2019); therefore, it is too early to assert that our model is optimal. The models should be validated in a real-world cohort. We hope to address these concerns in our future work.

In conclusion, the GC diagnostic and prognostic models established in the current study are low cost, highly sensitive, specific, and may facilitate accurate and individualized treatment for patients with GC.

## Data Availability

Publicly available datasets were analyzed in this study. This data can be found here: https://xena.ucsc.edu/. https://www.ncbi.nlm.nih.gov/geo/. http://ualcan.path.uab.edu/analysis.html.

## Supplementary Material

Supplementary TableS1 The methylation status of DMS: 1842 hypermethylation sites and 899 hypomethylation sites were screened from 27 normal samples and 443 GC samples.

## Glossary

GC: gastric cancer
DNAm: DNA methylation
DMS: DNA methylation sites
DMG: DNA methylation driver gene
TCGA: the cancer genome atlas
GEO: gene expression omnibus
MsigDB: molecular signatures database
LASSO: least absolute shrinkage and selection operator
FDR: false discovery rate
GSEA: gene set enrichment analysis
ROC: receiver operating characteristic
NPAS2: neuronal PAS domain protein 2
DAPK1: death associated protein kinase 1
CNN3: calponin 3
FGFR2: fibroblast growth factor receptor 2
PLEKHA5: pleckstrin homology domain containing A5
CEP290: centrosomalprotein290
CCDC69: coiled-coil domain containing 69
UBXN8: UBX domain protein 8
KDM4A: lysine demethylase 4A
AKR1B: aldo-keto reductase family 1 member B
RASSF2: ras association domain family member 2
KDELR3: KDEL endoplasmic reticulum protein retention receptor 3
CHRNB2: cholinergic receptor nicotinic beta 2 subunit
EGR1: early growth response 1
ARMC9: armadillo repeat containing 9
RPN1: ribophorin I
PDCD1: programmed cell death 1
CTLA4: cytotoxic T-lymphocyte associated protein 4
LAG3: lymphocyte activating 3
TIGIT: T cell immunoreceptor with Ig and ITIM domains
GZMB: granzyme B
TNF: tumor necrosis factor
EMT: epithelial-mesenchymal transition
CDH1: cadherin 1
TF: transcription factors.
